# Editorial: Disparities in Cancer Prevention and Epidemiology

**DOI:** 10.3389/fonc.2022.872051

**Published:** 2022-06-01

**Authors:** Fateme Montazeri, Hamidreza Komaki, Farnam Mohebi, Bahram Mohajer, Mohammad Ali Mansournia, Saeid Shahraz, Farshad Farzadfar

**Affiliations:** ^1^ Department of Ophthalmology and Vision Science, University of California, Davis, Sacramento, CA, United States; ^2^ Khoury College of Computer Sciences, Northeastern University, Boston, MA, United States; ^3^ Haas School of Business, University of California, Berkeley, Berkeley, CA, United States; ^4^ Department of Radiology and Radiological Science, School of Medicine, Johns Hopkins Medicine, Baltimore, MD, United States; ^5^ Department of Epidemiology and Biostatistics, School of Public Health, Tehran University of Medical Sciences, Tehran, Iran; ^6^ Institute for Clinical Research and Health Policy Studies, Tufts Medical Center, Boston, MA, United States

**Keywords:** cancer prevention, cancer epidemiology, disparities, inequality, gender disparities, racial disparities, socioeconomic disparities, population-specific

There were 23.6 million new cancer cases in 2019 in the world, causing 10 million deaths and 250 million disability-adjusted life years (1). The burden of the cancer has dramatically increased since 2010 such that cancer new cases, deaths, and disability-adjusted life years increased by 26.3%, 20.9%, and 16.0%, respectively, in 2019 (1). The largest percentage increases have occurred in the low and low-middle socio-demographic index quintiles, suggesting inequal distributions of cancer cases and burden in different populations. Therefore, not only we generally need to improve cancer prevention and control, but we should also aim to make efforts to address inequal burden of cancer among different groups of patients (1). To do so, Disparities in Cancer Prevention and Epidemiology Research Topic in Frontiers in Oncology journal attempted to understand the coordinates and causes of the existing disparities in cancer prevention and distribution in groups of patients with the goal of tackling the by means of of evidence-informed and population-specific policy making. **Table 1** provides a summary of the articles in this Research Topic.

**Table 1:** Summary of studies included in Disparities in Cancer Prevention and Epidemiology.

Authors	Title	Country of Origin	Aim/Purpose	Number of Participants	Summary of Result	Interpretation
Permuth et al.	Comparison of Radiomic Features in a Diverse Cohort of Patients with Pancreatic Ductal Adenocarcinomas	USA	Investigation of disparities between African American, Non-Hispanic Whites, and Hispanic/Latinx patients with pancreatic cancer based on radiomic tumor profile retrieved from pretreatment CT images	71	Multiple textural radiomics features were identified as being independently associated with poor prognosis among African American patients with PDAC.	There are biological differences in populations with different race and ethnicity that influence their outcome of cancer.
Dasgupta et al.	Access to Aboriginal Community- Controlled Primary Health Organizations Can Explain Some of the Higher Pap Test Participation Among Aboriginal and Torres Strait Islander Women in North Queensland, Australia Paramita	Australia	Investigation of regional differences in the utilization of ACCHO services for cervical screening, as well as variations in screening participation among Aboriginal and Torres Strait Islander women	1,107,233	Aboriginal and Torres Strait Islander women in North Queensland had a higher likelihood of being screened at ACCHOs than women in the rest of Queensland, adjusted for age and area.	Facilitating access to health services reduce regional disparities for cancer screening programs.
Petrick et al.	Racial Disparities and Sex Differences in Early- and Late-Onset Colorectal Cancer Incidence, 2001–2018	USA	Assessing early- and late-onset Colorectal Cancer incidence rates in the US	2,585,621	Blacks and American Indians/Alaska Natives had the greatest incidence of both early and late-onset Colorectal Cancer. Early-onset Colorectal Cancers were stable in terms of incidence, though neuroendocrine tumors were on the rise. Due to rising rates among Whites, the early-onset Colorectal Cancer difference between Blacks and Whites had narrowed.	Racial disparity in cancer may be rooted in inequality of health care administration policies, social determinants of health, and structural racism.
Jung et al.	Synergistic Effects of Genetic Variants of Glucose Homeostasis and Lifelong Exposures to Cigarette Smoking, Female Hormones, and Dietary Fat Intake on Primary Colorectal Cancer Development in African and Hispanic/Latino American Women	USA	Genomic assessment of insulin resistance as a key biologic mechanism underlying Colorectal Cancer carcinogenesis due to obesity	6,678	Intake of dietary polyunsaturated fatty acids and long-term exposure to female hormones may be important factors in mediating the racial gap in Colorectal Cancer incidence between African American and Hispanic American women.	Differences in modifiable and non-modifiable risk factors of cancers, such as diet, biological, and genetic characteristics of patients, might cause and increase disparities in burden of cancer if they are not addressed in educational and screening programs.
Hamdi et al.	Cancer in Africa: The Untold Story	USA	Identifying the most promising African preventative and treatment approaches	GLOBOCAN report	Based on the Human Development Index and the availability of medical equipment, different regions of Africa had different patterns of cancer incidence and mortality rates.	Paucity of facilities or screening programs cause cancer disparities in different African regions.
Wallace et al.	Preinvasive Colorectal Lesions of African Americans Display an Immunosuppressive Signature Compared to Caucasian Americans	USA	Investigation of possible racially different immunological markers in the early phases of Colorectal Cancer	95	African Americans compared to Caucasian Americans had a lower effector response capacity and an immunosuppressive ('cold') tumor environment.	Inherited carcinogenesis risk factors must be considered in screening program designing.
Mongiovi et al.	Genetic Variants in COX2 and ALOX Genes and Breast Cancer Risk in White and Black Women Jennifer	USA	Examining the links between COX2 and three ALOX gene variations and the risk of Breast Cancer in White and Black women	2,574	Variations in the COX2 and ALOX genes were associated with Breast Cancer and varied across White and Black women in subgroups based on their menopausal and Estrogen Receptor status.	Genetic differences must be considered in cancer preventive program.
Chan et al.	Cancer Screening Knowledge and Behavior in a Multi-Ethnic Asian Population: The Singapore Community Health Study Tyson	Singapore	Investigation of cancer screening enrollment rates and screening behavior in a multi-ethnic community	7,125	In Singapore, screening for cervical, breast, and colorectal cancers was correlated with higher educational level, higher household income, and being Chinese as compared to Malay ethnicity.	Socioeconomic status and ethnicity have a significant impact on cancer screening rate and can be tackled by cultural and educational strategies and facilitating screening programs.
Bellaiche et al.	Disparity in Access to Oncology Precision Care: A Geospatial Analysis of Driving Distances to Genetic Counselors in the U.S.	USA	Investigation of equity of access to genetic counselors on a nationwide level	4,813	Access to genetic counselors for patients with cancer varied by area, socioeconomic status, and cancer type in the US.	Inequality in access to healthcare services varied by regions and socioeconomic status leading to disparities in cancer prevention.
Simon et al.	A Review of Research on Disparities in the Care of Black and White Patients with Cancer in Detroit	USA	Summation of nearly 30 years of study on Black-White disparities in cancer incidence, care, and outcomes by investigators at the KCI's PSDR program	Review	Black cancer patients had a poorer prognosis due to racial inequalities in primary cancer site, comorbid medical conditions, treatment, and physician-patient communication.	Disparities in cancer outcome between black and white population might be caused by different factors ranging from almost non-modifiable biological traits to completely modifiable physician-patient. Socio-demographic and clinical differences could account for some of the observed disparities, but the influence of systemic effects of racism against Black people needs to be investigated as well.
Biddell et al.	Racial and Ethnic Differences in the Financial Consequences of Cancer- Related Employment Disruption	USA	Examining the disparities in the financial effects of employment disruption according to race/ethnicity	619	In comparison to Non-Hispanic White participants, Non-Hispanic Black and Hispanic/Latinx patients were more likely to report job-related income loss and changes in health insurance when suffering from cancer.	Disparities in cancer outcomes are not limited to precancerous stages; even after being diagnosed with cancer, there are other aspects such as financial disruption that exacerbates the existing disparities and need to be addressed.
Blackman et al.	Colorectal Cancer Screening Prevalence and Adherence for the Cancer Prevention Project of Philadelphia (CAP3) Participants Who Self-Identify as Black	USA	Investigation of Colorectal Cancer screening prevalence and adherence to national screening recommendations, as well as the link between birth region and Colorectal Cancer screening adherence, among a diverse Black population	357	Caribbean and African immigrants adhered to Colorectal Cancer screening at a higher rate than US-born Blacks.	Disparity in subgroups of black populations might reveal more fundamental aspects of inequality based on historical racism or immigration effects.
Nam et al.	Interactions Between Adiponectin- Pathway Polymorphisms and Obesity on Postmenopausal Breast Cancer Risk Among African American Women: The WHI SHARe Study	USA	Investigation of the interaction of genetic variants linked to adiponectin phenotype, obesity, and the risk of breast cancer in African American women	7,991	Obesity was a significant effect modifier for the association between SNPs and Breast Cancer risk in postmenopausal African American women.	A potential intervention to reduce disparities in cancer outcomes is to design cancer screening programs specific to populations with the goal of addressing their unique needs.
Pinheiro et al.	Endometrial Cancer Type 2 Incidence and Survival Disparities Within Subsets of the US Black Population	USA	Comparing incidence and survival patterns of Endometrial Cancer Type 2 among US Black ethnic groups: US-born Blacks, Caribbean-born Blacks, and Black Hispanics	24,387	The incidence and mortality of Endometrial Cancer Type 2 was higher in people of African descent. And the US-born Blacks, Caribbean-born Blacks, and Black Hispanics groups had substantial intra-racial differences.	Cancer disparities exist even within the race and ethnicity social categories. To tackle the barriers to access to cancer prevention programs, policies should be designed for each specific group of populations.

AA: African American, ACCHO: Aboriginal and Torres Strait Islander Community-Controlled Health organizations, CT: Computed Tomography, KCI: Karmanos Cancer Institute, PDAC: Pancreatic Ductal Adeno Carcinoma, PSDR: Population Studies and Disparities Research.

This Research Topic was established because although there are considerable number of effective and efficient preventive strategies for many types of cancers, still some populations are severely and unequally suffering from cancer. These preventive strategies and practices consist of, but are not limited to, preventing exposure to identified carcinogens, risk factor management, vaccination against cancer, screening for subclinical incidence, and early detection of the clinically present cancers. But these programs are not equally and equitably helping patients in different populations. A part of the unequal benefit of these interventions for different groups of patients is due to patients' biophysical attributes and their differences in the likelihood of developing cancer and the prognosis (2). Nevertheless, the existing disparities among patient populations are mainly caused by inequalities in cancer prevention and care and other related aspects of healthcare rather than biological differences in patients. The followings depict the steps of care in which different factors cause the discussed disparities.

The first stage of cancer prevention is individuals becoming aware that if they belong to high-risk groups for a cancer, they need to be screened for it. Therefore, a potential point of intervention to address inequalities in cancer prevention and care is to increase public awareness of screening programs or vaccination and emphasize their importance in groups of patients who are not appropriately utilizing preventive and screening services. The strategies and interventions should be designed to create a comprehensive understanding of screening in populations according to their differential background, education, gender, race, ethnicity, culture, and socioeconomic status. And these interventions should be tailored to specific needs of each patient group. As an example, and in this Research Topic, Chan et al. showed that the ever-screened rates for cervical and breast cancer improved in parallel with increasing the screening knowledge in Singapore (cervical, 70.1 vs. 77.1%; breast, 54.2 vs. 75.2%), indicating the role of awareness in preventive service utilization. However, the outcome of increasing people’s knowledge varied depending on their socioeconomic status and ethnicity which directly supports the argument that each population should have their own intervention uniquely designed.

Having perceived the need, the second stage in cancer prevention is utilizing the preventive healthcare service. Regarding preventive care utilization, we first need to understand where the disparities are coming from and what the barriers to care equity are. Differences in perceived benefits and costs of preventive care is one of the factors that cause unequal access to care. Individuals make the decision to utilize a cancer prevention service by comparing the perceived costs and benefits of a service. And these perceptions are influenced by different factors including their socioeconomic status and financial support (3). Therefore, the costs and benefits of services are not just a matter of objective assessments. Services with exactly similar estimated costs could extremely differ in the cost that patients in different bio-socio-economic groups perceive them. Chan et al. supported this concern and reported that poor understanding of the screening procedure, fear of pain and diagnosis, and scheduling difficulty limit preventive service utilization because these factors increase the patients' perceived cost of screening. To elaborate, a group of patients perceived the preventive service to be more costly and less beneficial than others not because the costs of the service were higher for them or they objectively would benefit less from the care. But because that group of patients did not have appropriate familiarity with the preventive care and the fear of pain, for example, increased their perceived cost.

By studying and identifying what contributes to the perceived costs and benefits of screening in different populations, policies could be particularly designed for each population and effectively address their unique needs. As an illustration, the population in Chan et al. study would benefit most from interventions that address their fear and knowledge of screening while Dasgupta et al. study population need physically closer healthcare provision centers to decrease their perceived cost of care. No matter how much we decrease the fear of pain in the population studied by Dasgupta et al., they still cannot afford to travel the distance and utilize the care. Taken together, the goals of each promising intervention such as social network-based policies, could only be realized if the policy incorporates unique features of the patients' social lives and understand their special needs and barriers (4).

As we previously and slightly discussed, the percevied benefits and costs of care also depend on the accessibility and quality of the preventive care. Human resources, such as professional health care workers, healthcare facilities, and access to necessary technologies are important for cancer patients’ preventive care and they must be equitably distributed. Namely, in this Research Topic, Hamdi et al. showed that there is a huge gap in access to relatively simplest types of preventive care in different populations. They reported that in Western, Eastern, and Central African regions, the higher mortality rate of the most preventable cancers like breast, cervical, and prostate cancer is in tandem with the paucity of facilities or screening programs compared to Northern and Southern settings. And it is worth noting that the preventable services of these cancers are among the most easily accessible and affordable types of care in their setting. Bellaiche et al. also supported this notion by showing that access to a high-quality genetic consult for precision medicine depends on where a patient lives in the United States, indicating that even in a developed country not all patients face similar costs of care. And finally, Dasgupta et al. showed that a great proportion of the existing disparities in preventive care in indigenous women could be addressed/resolved by improving their access to primary health care, supporting the importance of understanding the unique needs of each group of patients.

Population-specific policy design is also important for patients. As an instance populations differ in how much burden their diagnosed cancer could cause them. For example, in some instances, the higher burden of cancer in a group of patients is due to lower acceptability of cancer-related programs and, thus, increasing the acceptability of the provided healthcare services could help to narrow the gap in burden of cancer for different patients. In agreement with this, Chan et al. showed that patients’ and physicians’ linguistic and ethnic concordance significantly improved healthcare service efficiency. Additionally, some populations are hit harder by cancer and require more protecting interventions. As an illustration, Biddell et al. showed that cancer’s cost is different for patients of the non-Hispanic black race, compared to patients of the non-Hispanic white race. Black patients in their study were more likely to lose their income and insurance after being diagnosed with cancer. And while non-Hispanic black patients were diagnosed with more aggressive cancers that required more expensive treatment, their employment flexibility and income were significantly limited compared to non-Hispanic white patients.

As of now, we realized how different factors in each step of healthcare utilization could have contributed to the existing disparities. Nevertheless, some might argue that a great proportion of disparities are caused by factors such as age, gender, race, and ethnicity of patients that are non-modifiable. We argue that healthcare systems can still ameliorate the disparities in cancer prevention and care through the modifiable factors or providing more and specifically designed care to those who are more likely to experience higher cancer burdens due to non-modifiable risk factors (Nam et al., Jung et al.). The changes that target the modifiable contributors to disparities in cancer burden include the inequalities that are rooted in factors such as, but not limited to, racioethnic discriminations. For example, Pinheiro et al. and Blackman et al. showed that there are disparities in cancer incidence and screening even among the Black population of the US that might be due to some historical racism or immigration effects. This study, per se, enlightens that racism, an example of a modifiable factor, could be used as a point of intervention to address disparities in cancer burden. The modifiable factors could also consist of biophysical conditions of patients. For example, Simon et al. showed that chronic kidney diseases, as preventable comorbidities, were more prevalent at the time of diagnosis and had a more significant adverse impact on renal cell carcinoma incidence in black patients than in white patients. Therefore, by designing prevention strategies that target chronic kidney diseases in black patients, we could decrease the black patients' burden of renal cell carinoma which is higher than white patients. And as previously discussed, even for non-modifiable factors, decision makers could design policies to more intensively help patients with a higher bio-physical probability of being diagnosed with cancer or suffering from more aggressive cancers with the hope of closing the gaps of cancer's burden between different populations. Accordingly, Simon et al., Wallace et al., and Mongiovi et al. showed that Black women in the United States are more likely to be diagnosed with more aggressive breast tumors or different immune responses in colorectal cancer, resulting in a higher incidence and mortality rate. Permuth et al. also demonstrated that some specific radiologic biomarkers for pancreatic cancer have only been reported in African Americans, not non-Hispanic white Americans or Hispanic/Latinx, indicating racial biological variations. To provide an example of what the goal of this Research Topic is and how it could be realized, we argue that these two studies suggest a potential point of intervention to address inequalities in cancer burden: more aggressively screening Black women for breast cancer and taking extra care of Black women with diagnosed breast cancer and all African Americans with pancreatic cancer. Therefore, a part of the gap in cancer burden could be closed by deliberately providing more care to more vulnerable populations. Taken together, care for cancer prevention and burden has multiple stages and each could be a point of intervention to control modifiable factors in more suffering patients or provide extra attention and support to patients with non-modifiable factors that make them more vulnerable to cancer and cause them to experience higher burdens.

All in all, this Research Topic presented a non-comprehensive but enlightening collection of research studies on the disparities in cancer prevention and epidemiology and it shed light on the aspects of cancer care that are potential fields for further exploration. Therefore, the reported results could be directly used for popultion-specific and effective intervention designs. Or the studies could serve as a guide for future investigations. This is particularly important because this Research Topic revealed that there is an absolute need for more research that provides thorough understanding of the life course of cancer patients in different biological, social, and economic groups. This information could help policy makers and researchers to understand what the contributing factors to the existing inequalties in cancer prevention, epidemiology, and burden are and how they could tackle these inequalities through population-specific studies and policy designs.

## Author Contributions

FMon and HK drafted the manuscript and incorporated the ideas of all authors. BM provided comments and approved of the final version. FMoh devised the idea, supervised the drafting, and finalized the manuscript. All authors contributed to the article and approved the submitted version.

## Conflict of Interest

The authors declare that the research was conducted in the absence of any commercial or financial relationships that could be construed as a potential conflict of interest.

## Publisher’s Note

All claims expressed in this article are solely those of the authors and do not necessarily represent those of their affiliated organizations, or those of the publisher, the editors and the reviewers. Any product that may be evaluated in this article, or claim that may be made by its manufacturer, is not guaranteed or endorsed by the publisher.
